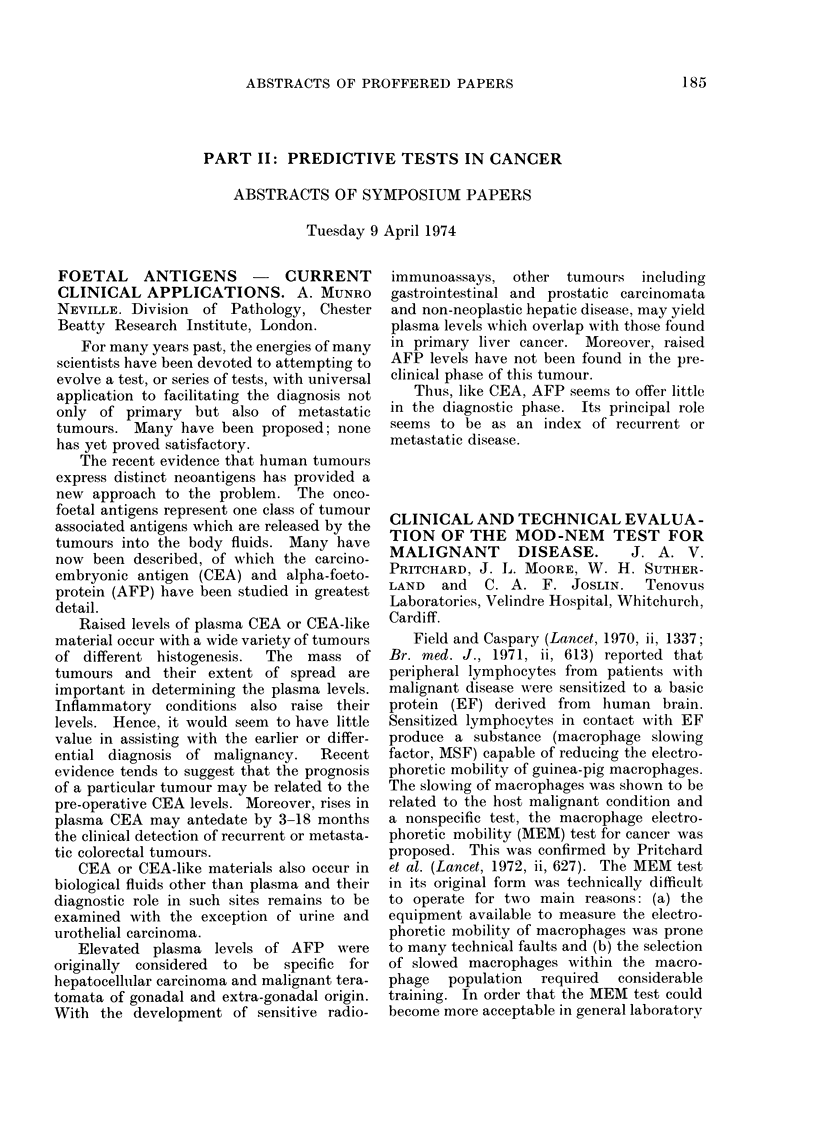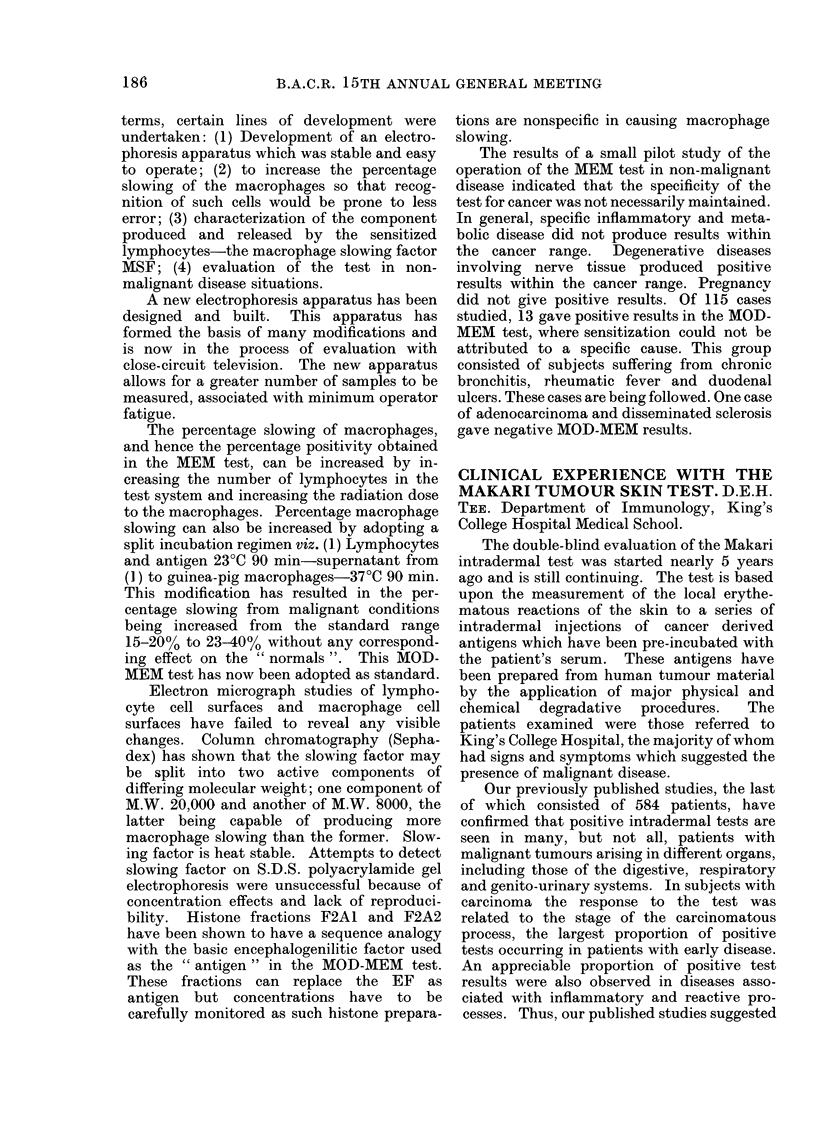# Proceedings: Clinical and technical evaluation of the MOD-NEM test for malignant disease.

**DOI:** 10.1038/bjc.1974.173

**Published:** 1974-08

**Authors:** J. A. Pritchard, J. L. Moore, W. H. Sutherland, C. A. Joslin


### CLINICAL AND TECHNICAL EVALUATION OF THE MOD-NEM TEST FOR MALIGNANT DISEASE.

J. A. V. PRITCHARD, J. L. MOORE, W. H. SUTHERLAND and C. A. F. JOSLIN. Tenovus Laboratories, Velindre Hospital, Whitchurch, Cardiff.

Field and Caspary (*Lancet*, 1970, ii, 1337; *Br. med. J.*, 1971, ii, 613) reported that peripheral lymphocytes from patients with malignant disease were sensitized to a basic protein (EF) derived from human brain. Sensitized lymphocytes in contact with EF produce a substance (macrophage slowing factor, MSF) capable of reducing the electrophoretic mobility of guinea-pig macrophages. The slowing of macrophages was shown to be related to the host malignant condition and a nonspecific test, the macrophage electrophoretic mobility (MEM) test for cancer was proposed. This was confirmed by Pritchard *et al.* (*Lancet*, 1972, ii, 627). The MEM test in its original form was technically difficult to operate for two main reasons: (a) the equipment available to measure the electrophoretic mobility of macrophages was prone to many technical faults and (b) the selection of slowed macrophages within the macrophage population required considerable training. In order that the MEM test could become more acceptable in general laboratory terms, certain lines of development were undertaken: (1) Development of an electrophoresis apparatus which was stable and easy to operate; (2) to increase the percentage slowing of the macrophages so that recognition of such cells would be prone to less error; (3) characterization of the component produced and released by the sensitized lymphocytes—the macrophage slowing factor MSF; (4) evaluation of the test in non-malignant disease situations.

A new electrophoresis apparatus has been designed and built. This apparatus has formed the basis of many modifications and is now in the process of evaluation with close-circuit television. The new apparatus allows for a greater number of samples to be measured, associated with minimum operator fatigue.

The percentage slowing of macrophages, and hence the percentage positivity obtained in the MEM test, can be increased by increasing the number of lymphocytes in the test system and increasing the radiation dose to the macrophages. Percentage macrophage slowing can also be increased by adopting a split incubation regimen *viz.* (1) Lymphocytes and antigen 23°C 90 min—supernatant from (1) to guinea-pig macrophages—37°C 90 min. This modification has resulted in the percentage slowing from malignant conditions being increased from the standard range 15–20% to 23–40% without any corresponding effect on the " normals ". This MOD-MEM test has now been adopted as standard.

Electron micrograph studies of lymphocyte cell surfaces and macrophage cell surfaces have failed to reveal any visible changes. Column chromatography (Sephadex) has shown that the slowing factor may be split into two active components of differing molecular weight; one component of M.W. 20,000 and another of M.W. 8000, the latter being capable of producing more macrophage slowing than the former. Slowing factor is heat stable. Attempts to detect slowing factor on S.D.S. polyacrylamide gel electrophoresis were unsuccessful because of concentration effects and lack of reproducibility. Histone fractions F2A1 and F2A2 have been shown to have a sequence analogy with the basic encephalogenilitic factor used as the " antigen " in the MOD-MEM test. These fractions can replace the EF as antigen but concentrations have to be carefully monitored as such histone preparations are nonspecific in causing macrophage slowing.

The results of a small pilot study of the operation of the MEM test in non-malignant disease indicated that the specificity of the test for cancer was not necessarily maintained. In general, specific inflammatory and metabolic disease did not produce results within the cancer range. Degenerative diseases involving nerve tissue produced positive results within the cancer range. Pregnancy did not give positive results. Of 115 cases studied, 13 gave positive results in the MOD-MEM test, where sensitization could not be attributed to a specific cause. This group consisted of subjects suffering from chronic bronchitis, rheumatic fever and duodenal ulcers. These cases are being followed. One case of adenocarcinoma and disseminated sclerosis gave negative MOD-MEM results.